# Regulation of Immune Responses by Histone Deacetylase Inhibitors

**DOI:** 10.5402/2012/690901

**Published:** 2012-03-18

**Authors:** Paul V. Licciardi, Tom C. Karagiannis

**Affiliations:** ^1^Allergy and Immune Disorders Group, Murdoch Childrens Research Institute, Melbourne, VIC 3052, Australia; ^2^Department of Paediatrics, University of Melbourne, Melbourne, VIC 3052, Australia; ^3^Epigenomic Medicine Laboratory, Baker IDI Heart & Diabetes Institute, Melbourne, VIC 3004, Australia; ^4^Department of Pathology, University of Melbourne, Parkville, VIC 3052, Australia

## Abstract

Both genetic and epigenetic factors are important regulators of the immune system. There is an increasing body of evidence attesting to epigenetic modifications that influence the development of distinct innate and adaptive immune response cells. Chromatin remodelling via acetylation, methylation, phosphorylation, and ubiquitination of histone proteins as well as DNA, methylation is epigenetic mechanisms by which immune gene expression can be controlled. In this paper, we will discuss the role of epigenetics in the regulation of host immunity, with particular emphasis on histone deacetylase inhibitors. In particular, the role of HDAC inhibitors as a new class of immunomodulatory therapeutics will also be reviewed.

## 1. Introduction

It is clear that modulation of gene transcription is an essential component of many biological processes. The onset of many pathological conditions such as cancer and chronic inflammation often results from aberrant gene transcription. Integral to this process are epigenetic factors involving two critical enzymes, histone acetyltransferases (HAT) and histone deacetylases (HDAC). These produce posttranslational modifications on histone proteins and result in changes to chromatin structure and function [1, 2]. While HAT serves to acetylate the N-terminal histone tail, conferring a “relaxed” chromatin structure that allows transcriptional activation, HDAC has the opposite effect and represses transcription through tightening of the chromatin structure, excluding accessibility of transcription factors and other regulatory proteins to bind DNA and therefore the ability to influence gene expression [3]. Activated transcription factors (e.g., NF-*κ*B, AP-1, STAT) bind to promoter regions on genes leading to the recruitment of CREB-binding protein (CBP)/adenoviral protein E1A (p300) and other coactivation proteins to form a transcriptional initiation complex, leading to histone acetylation and gene transcription [4]. It is widely recognised that aberrant gene transcription resulting in either HAT inactivation or HDAC overexpression can lead to increased tumour cell proliferation (a major mechanisms of oncoproteins) as well-regulating proinflammatory responses [5]. In humans, a total of 18 HDAC members have been identified and characterized to date, falling into four classes: class I (HDAC1,2,3,8), class II (HDAC4,5,6,7,9,10) and class III (Sir2-like deacetylase sirtuins1-7), and class IV (HDAC11) [6, 7]. Both class I and class II are involved in regulating proinflammatory responses as well as cell proliferation and differentiation while the functions of class III HDACs are not fully understood. Class IV HDAC11 has been recently characterized as enzymes modulating the balance between immunity and tolerance [8].

## 2. Histone Deacetylases and Immunity

The role of HDACs in the epigenetic regulation of innate and adaptive immunity is of significant interest. Understanding the level of HDAC expression within the immune system would assist the development of more targeted immunomodulatory therapeutic strategies. The multitude of HDAC isoforms may elicit further control over the complex immune response dynamics during effector cell functioning. The molecular mechanism(s) by which HDACs mediate their biological activity are diverse, either by direct inhibition of gene transcription or indirectly through modulation of nuclear transcription factors such as NF-*κ*B.

Transcription factors such as NF-*κ*B, GATA-3, T-bet, and Foxp3 are integral to the development of immunity. For the NF-*κ*B family proteins, p65 (RELA) and p50 (NF*κ*B1), interaction with HDAC1 and HDAC2 has wide-ranging effects on the immune system [9]. While HDAC1 and HDAC2 have been shown to bind with the NF-*κ*B family corepressor protein p65 and downregulate NF-*κ*B-mediated gene transcription, in unstimulated cells, this is also achieved by HDAC1 interactions with another NF-*κ*B corepressor, p50 [10–12]. Upon activation, this complex is displaced by phosphorylated p65 and the transcriptional coactivator, CBP, increasing gene transcription. HDAC enzymes are therefore critical in the balance of inflammatory responses mediated by IL-6, IL-8, IL-1*β*, and GM-CSF by regulating the histone acetylation status of NF-*κ*B and AP-1. Evidence also suggests that NF-*κ*B activation status is also dependent on HDAC3 in the regulation of target gene promoter hyperacetylation (e.g., I*κ*B-*α*, IL-2, IL-6) [13]. Moreover, maintenance of NF-*κ*B acetylation occurs following cytosolic I*κ*B-*α* binding to HDAC1 and HDAC3, preventing inactivation of NF-*κ*B transcription [14]. HDAC3-mediated deacetylation of p65 results in shuttling of the NF-*κ*B complex from the nucleus to the cytoplasm, thus controlling the proinflammatory response [11]. Oxidative stress producing reactive oxygen species in the context of cigarette smoking, chronic obstructive pulmonary disease, and air pollutants are all known to inhibit HDAC activity, thereby enhancing transcription of proinflammatory genes via NF-*κ*B and exacerbating these activities [15, 16]. Similar HDAC effects have been reported in animal models of trauma/haemorrhage in the repression of proinflammatory responses [17]. More recently, interest in the role of environmental exposures during pregnancy on epigenetic changes in the fetus that either silence or activate gene expression may provide clues to critical pathways leading to disease susceptibility and pathogenesis.

HDAC activity also inhibits dendritic cell (DC) function by repressing the acetylation of histone and nonhistone proteins such as STAT-3 [18]. This negative regulation of DC function has important implications in the induction of effector immunity. The class I HDAC, HDAC1, has been previously reported to repress TLR-inducible genes as well as the IL-12p40 promoter although this is not completely understood since recent studies have shown upregulated IL-12p40 gene in DCs by HDACs [19], possibly by posttranslational modifications that enhance transcription factor recruitment to promoter regions. It was proposed that certain HDACs could reverse the acetylation of nonhistone proteins such as transcription factors p53, GATA, SMAD7, and NF-*κ*B.

Epigenetic mechanisms such as DNA methylation and histone modifications have been associated with the transcription and expression of the Foxp3 gene [20] and were involved in Foxp3+ Treg differentiation into IL-17 producing cells. In addition, the finding that histone H3 and H4 acetylation was associated with a conserved region within the Foxp3 locus for CD4+CD25+ Treg but not conventional CD4+CD25− T cells, similar to that for DNA methylation, suggests an important role for epigenetics in maintaining Foxp3 expression. Dysregulation of this response may favour enhanced HDAC activity which would destabilize this process and may lead to exacerbated immunopathologies. In addition, both Th2 cytokine and GATA-3-driven T-cell responses as well as Th17 cells are also activated following redox-induced HDAC inhibition [21–23]. The Th17 phenotype is partly controlled by TGF-*β* (via SMAD signalling) which inhibits both T-bet and GATA-3 and subsequent Th1/Th2 cytokine production [24, 25]. A new member of the HDAC family, HDAC11 (class IV), has recently been implicated as a critical molecular target in the immune system that directs activation or tolerance [8]. Experimental evidence demonstrated that HDAC11 represses IL-10 gene expression in human and murine APCs leading to immune activation of previously tolerant CD4+ T cells [26]. Conversely, APCs lacking in HDAC11 were shown to upregulate IL-10 gene expression and can tolerize antigen-specific T cells. The manipulation of HDAC11 by specific HDAC inhibitors represents a novel approach in the treatment of a variety of immunological conditions as well as in the immunotherapy of cancer.

HDAC enzymes could also help control Th1 and Th2 differentiation of naive CD4+ T cells by reversing the hyperacetylation of histones 3 and 4 at the IFN-*γ* promoter [27]. This would be useful since such epigenetic changes are stably inherited by fully differentiated effector T cells [22]. It has been reported that HDACs interact with regulators of MHC class II gene activation (e.g., CIITA) and act as molecular switches to turn off this process [28]. The use of bioinformatics has helped identify conserved noncoding regions in the IFN-*γ* gene that are associated with increased Th1-specific H4 acetylation as well as locate T-bet binding due to the presence of cluster regions containing transcription factor binding sites for NF-*κ*B, T-bet, GATA-3, and STAT-4/6. The HDAC-mediated control of IFN-*γ* Th1 effects may be required in the control of certain immunological conditions. GATA-3 helps maintain repression of Th1 effects by binding to HDAC enzymes which then interacting with the IFN-*γ* gene while allowing stable Th2 differentiation [29]. The fact that T-bet can bind directly with GATA-3 to inhibit this activity ensures a balance between these Th lymphocyte functions [30].

## 3. Histone Deacetylase Inhibitors as Therapeutic Immunomodulators

**Figure 1:**
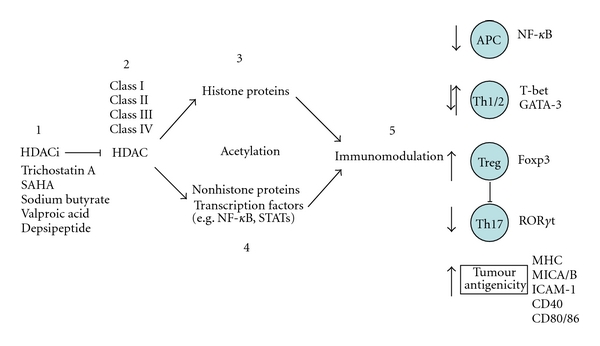
Schematic representation of HDACi immunomodulation. (1) HDACi such as Trichostatin A and SAHA suppress the activity of class I/II HDAC enzymes (2). This reverses the acetylation status, leading to (hyper)acetylation of both (3) histone and (4) nonhistone proteins such as nuclear transcription factors. Together, chromatin remodelling and immune gene expression is altered and, in the context of the immune system, can lead to immunomodulation (5). Among the pleiotropic activities of HDACi, activation of Treg cells limits the extent of inflammatory-mediated tumourigenesis as well as the development of Th17 cells. HDACi also directly inhibit the activity of Th1 cells mainly by repression of the Th2 regulator, GATA-3. Inhibition of proinflammatory APC function is also mediated by HDACi by modulating NF-*κ*B activation status. These properties of HDACi are also important in the immunosurveillance of cancer by upregulating specific markers that enhance tumour antigenicity and targeted immune system-mediated cytotoxicity CD8+ T cells and NK cells.

The clinical use of HDAC inhibitors (HDACi) has been mainly focused on the treatment of cancer based on the documented antiproliferative activities involving regulation of gene expression, cell cycle arrest, apoptosis, and antiangiogenesis effects [2, 31–33]. Since the expression of HDACs influences the development and differentiation of immune responses, the identification of a variety of molecules able to inhibit particular HDAC enzymes (HDACi) offers an exciting and novel approach to the treatment of immune-mediated diseases (Figure 1). More specifically, HDACi with reported effects on autoimmune disease, transplantation, and infection will be discussed here in after.

HDAC inhibitors (HDACi) are a potential source of novel immunomodulatory drugs aimed at treating a wide range of diseases. In particular, the maintenance of an anergic state is associated with repression of the IL-2 gene promoter region [34, 35]. This occurs as a result of epigenetic imprinting that can be inherited over multiple cell division cycles [36]. CD4+ T-cell activation requires antigenic and costimulatory signals and is dependent on histone acetylation chromatin remodelling at the IL-2 promoter [37]. However, anergy results from the lack of costimulation as well as histone hypoacetylation of both IL-2 and IFN-*γ* promoters where HDAC activity is thought to maintain this anergic phenotype. Inhibition of HDAC activity in this context restores histone acetylation in conjunction with reduced Ikaros expression (that interacts with corepressor complexes) to relieve this anergic state. The potential use of HDACi in the treatment of immune disorders or as adjuvants that direct the generation of specific immune phenotypes has significant implications in the design of new pharmacologicals or prophylactic drug design. Increased understanding of the biological properties of HDACi will help delineate how these molecular targets can “switch” the immune system in response to any given challenge.

Using the pan-HDACi, LAQ824, both upregulation and downregulation of specific innate immune genes were found in TLR4 (LPS) activation of APCs such as monocytes and DCs [38]. HDACi-treated murine DCs cocultured with OVA-stimulated CD4+ T cells selectively inhibited IFN-*γ* by Th1 cells but had no effect on Th2 cytokine production. Similarly, the addition of another HDACi, sodium butyrate, a short-chain fatty acid, to DC cultures also inhibits CD1 expression (involved in presentation of lipid antigens) but not CD83, CD86, or MHC molecules at the protein and mRNA level as well as Th1 cytokine responses [39]. Using the well-characterized class I/II HDACi, Trichostatin A (TSA), CD4+ T-cell proliferation was inhibited with a concomitant increase in caspase-independent apoptosis in primary murine T cells [40]. The biological activity TSA was also demonstrated by suppression of IL-2 gene expression and NF-*κ*B protein levels. A similar inhibitory effect on human T cells was observed following HDACi treatment associated with inhibition of IL-2 secretion by activated T cells, reduced CD154 (CD40L), CD25 but not CD69 expression, as well as a reduction in c-myc expression [41]. These immunomodulatory activities of HDACi suggest an important role as novel therapeutic agents in the amelioration of immune and inflammatory-mediated conditions.

Differentiation and function of DCs were impaired following treatment with the HDACi, valproic acid (VPA), and MS-275 [42]. This was based on the downregulation of the costimulatory markers CD1a, CD80, CD83, and the CD54 adhesion marker even in the presence of the TLR3 ligand, poly (I-C) known to induce the expression of these markers under normal conditions. The effects of VPA and MS-275 on altered DC function were demonstrated with impaired stimulatory capacity of allogeneic lymphocytes. These HDACi effects were mediated through altered NF-*κ*B and interferon regulatory factor (IRF) signalling. Using an alternative HDACi, the fungal metabolite apicidin, Th1 polarization of murine bone marrow-derived DCs stimulated with LPS was suppressed [43]. Apicidin attenuated the secretion of IL-12 and proinflammatory cytokines such as IL-6 and TNF-*α* as well as IFN-*γ* by T cells that was dependent on NF-*κ*B inhibition in these cells. A protective effect has also been established for TSA by blocking PBMC proliferation and abrogating IFN-*γ* production by Th1 cells leading to apoptosis [44]. This provides a mechanism by which HDACi may be exploited for immunotherapeutic use.

The ability of HDACi to modulate the regulatory immune response has been successful. Treatment of mice with TSA augments natural Foxp3+ Treg cells as well as Treg gene expression and suppressive function [45]. TSA treatment of these Treg cells also increased mRNA levels of Foxp3, CTLA4, GITR, and IL-10 that are important in this response. The TSA activity was associated with acetylation of Foxp3 itself as well as histones contained within Tregs further evidence that nonhistone proteins are targets for HAT/HDAC enzymes [46]. Moreover, the suppressive Treg function was HDAC9 dependent, providing novel insights into the role of HDAC9 in Foxp3-mediated transcriptional repression [47] and the potential targeting of this interaction in the manipulation of immunological responses. Other studies have also demonstrated the capacity of TSA to induce the differentiation of Treg cells from naive T cells via the epigenetic enhancement of Foxp3 expression [48]. The regulatory activity of histone deacetylase inhibitors such as TSA and SAHA was also shown to significantly inhibit the secretion of Th1 and Th17 cytokines (IL-12 and IL-23, resp.) as well as the selective inhibition of Th1-attracting chemokines (CXCL9, 10, 11) by DCs in an LPS/IFN-*γ* inflammatory model [49]. Indeed, inflammation-induced tumour development as observed in experimental colitis disease models can be abrogated by HDACi through modulation of regulatory immune responses [50]. Since Th17 cells in particular have important roles in the defence against bacterial infections as well as in the protection from autoimmune diseases, these effects of HDACi provide further justification for the further investigation of these agents in the control of immune responses.

## 4. Histone Deacetylase Inhibitors and Transplantation

The role of HDACi in promoting allograft survival and transplant tolerance is dependent on critical immune effector functions. Trichostatin A has been reported to enhance allograft tolerance in synergy with low-dose rapamycin treatment that was dependent on the development of intragraft Foxp3+ Treg cells [51]. In mouse models of cardiac and islet transplantation, increased survival times past 100 days were observed following HDACi treatment with maintenance of tissue and cellular integrity after transplantation [52]. TSA also enhances the production of Foxp3+ Treg with suppressive function [53] associated with increased histone H3 and Foxp3 acetylation *in vivo *[54]. In a model of experimental graft-versus-host disease (GVHD) using murine allogeneic bone marrow transplantation (BMT), pretreatment of bone marrow-derived DCs with SAHA (a derivative of TSA) upregulated expression of the DC-suppressor, indoleamine 2,3-dioxygenase (IDO) leading to reduced GVHD [55]. This effect for SAHA was associated with reduced DC-stimulated expression of CD40 and CD80 *in vitro *as well as reduced TLR-mediated proinflammatory cytokine secretion. Furthermore, Tregs isolated from HDAC6-deficient mice had increased FoxP3 and IL-10 mRNA expression compared to wild-type mice, and these Tregs exhibited increased suppressive activity suggesting an important role for this enzyme [56]. Indeed, pharmacological inhibition of HDAC6 using the isoform-specific inhibitors, tubacin, and tubastatin A produced similar results and induced long-term cardiac allograft survival in mice. Similarly, the effect of HDACi such as SAHA was also found to increase Treg suppressive activity that was found to correlate with CTLA-4 levels rather than FoxP3 [57]. Evidence for the role of HDAC11 in transplant survival has only recently emerged, with inhibition of this enzyme using short hairpin RNA interference (RNAi) reducing HDAC11 mRNA and protein levels in liver tissue with a concomitant increase in IL-10 following liver transplantation in rats [58]. These changes were associated with increased survival rates after 1 week, and the effects on IL-10 were thought to be safer than conventional immunosuppressant therapy. Modulation of HDAC11 expression was detected in liver Kupffer cells (macrophages) after transplantation [59], providing a mechanism for tolerance induction. Therefore, HDACi with immunological activities represent promising alternatives or adjuncts to current treatment modalities, and clinical trials involving the use of HDACi will be important.

## 5. Histone Deacetylase Inhibitors and Infection

The effectiveness of HDACi for the treatment of infections has been mostly studied in the context of HIV. Treatment of HIV by Highly Active Antiretroviral Therapy (HAART) does not completely eradicate latently infected cells that are a source for viral reactivation and increased viral load [60]. Several *in vitro *studies have reported the ability of HDACi to induce viral replication under latency conditions suggesting that combination therapy consisting of HAART and HDACi may augment this response [61–63]. Using the HDACi, valproic acid (VPA), or SAHA in combination with an NF-*κ*B inducer, prostratin, a higher reactivation of HIV-1 production was observed in latently infected U1 and J-Lat cell lines as well as in CD8+-depleted PBMCs from HIV-1-infected patients receiving HAART where viral load could not be detected compared to either HDACi alone [60, 62]. The ability of VPA to increase and prolong prostratin-induced NF-*κ*B DNA binding was postulated as being part of the regulatory NF-*κ*B pathway modulated by histone and/or nonhistone changes [64]. Indeed, VPA reversed the repressive activity of the nuc-1 nucleosome [65] (which is immediately downstream of the HIV-1 transcription promoter) and induced the acetylation of histone H4. Furthermore, HDAC3-specific inhibition by VPA was necessary to activate latent HIV in Jurkat cells *in vitro, *although this effect was only moderate [66]. Treatment with SAHA in an LPS mouse model of septic shock improved survival rates associated with increased liver anti-inflammatory IL-10 levels while decreasing proinflammatory IL-6 and MAP kinase production [67]. The biological activities of HDACi in the context of malaria and fungal diseases have also been examined with limited success [68].

In contrast, HDACi treatment has also been shown to impair host defense against bacterial infections. Recent studies have shown that HDAC inhibition by TSA, SAHA, and VPA can impair innate immune responses to TLR agonists by reducing the expression of genes associated with microbial sensing (C-type lectins, adhesion molecules, and others) as well as a range of cytokine and chemokine genes [69], thereby increasing susceptibility to infection. Interestingly, while VPA increased mortality from lethal *C. albicans *or *K. pneumonia *infection in mice, it increased survival in mouse models of toxic shock. Reduced killing of *E. coli *and *S. aureus *was also observed following *in vitro *treatment of murine macrophages with TSA and VPA, with impaired phagocytosis and reactive oxygen and nitrogen species generation [70]. Together, these data reveal the complex nature of HDACi effects and highlight the need for more studies in this context.

## 6. Histone Deacetylase Inhibitors and Immune-Mediated Disease

Emerging data on the experimental properties of HDACi have centred on immune-mediated diseases such as immunodeficiency conditions and autoimmunity. Many immunodeficiency conditions develop early in life and persist for many years into adulthood, often with limited treatments. New therapies that improve clinical outcomes will therefore be of significant advantage. The HDACi VPA was shown to significantly reduce lymphoproliferation in a murine model of autoimmune lymphoproliferative syndrome (ALPS) [71]. This is a condition characterized by lymphadenopathy, splenomegaly, hypergammaglobulinemia, elevated IL-10, and accumulation of double-negative T cells [72–74] associated with mutations in the *Fas *gene that is critical for lymphocyte homeostasis and peripheral immune tolerance [75]. VPA and another HDACi, depsipeptide, induced PBMC apoptosis in healthy individuals and ALPS patients *in vitro*, although depsipeptide was associated with reduced bone marrow cellularity [71]. In addition, VPA induced histone acetylation in splenocytes that persisted in the absence of serum VPA. Similar results have also been reported for TSA [76]. It is believed that the clinical activity of HDACi in ALPS patients may be more effective due to a lack of a functional extrinsic apoptosis pathway.

The role of HDACi in the treatment of multiple sclerosis (MS) was identified by early studies indicating that neuronal traits may be modulated by histone acetylation of neuronal genes [77]. The specific repressor protein, repressor element 1 silencing transcription factor (REST), is important since dysregulated REST contributes to neuronal loss [78]. Corepressor complexes of REST and HDACs have been isolated and recruitment of HDACs represses gene expression, with class I and II HDACs being critical for neuron survival and differentiation [79, 80]. In MS, HDACi have been shown to upregulate MHC class II genes since HDAC1 and 2 repress this gene expression [81, 82]. However, HDACi have been shown to ameliorate MS pathogenesis by preventing IFN-*γ*-induced B7.1 upregulation and enhancing B7.2 expression on T cells leading to a Th2 phenotype shift [83]. Furthermore, the therapeutic effects of HDACi may be associated with matrix metalloproteinase (MMP) expression since this is important in the inflammatory cell infiltration observed in MS. While the HDACi, sodium butyrate, increased MMP-9, another HDACi, apicidin, had no such effect while also inhibiting MMP-2 activity *in vitro *[84, 85]. Using the murine model of MS, experimental autoimmune encephalomyelitis (EAE), the HDACi TSA reversed the myelin-oligodendrocyte-glycoprotein- (MOG-) induced EAE via proinflammatory cytokine repression as well as histone hyperacetylation and inhibition of axonal apoptosis by caspase-dependent mechanisms [86].

In another model of autoimmune disease, HDAC inhibition using TSA and SAHA reduced the IFN-*γ* and IL-1*β*-mediated beta-cell destruction and reduced insulin secretion characteristic of type 1 diabetes mellitus *in vitro *[87]. In this example, inhibition of NO and beta cell apoptosis was revealed and was NF-*κ*B dependent, further indicating the clinical utility of HDACi in the protection from inflammatory-mediated diseases. The utility of HDACi in the treatment of inflammatory conditions also extends to asthma and other allergic diseases. In one study, TSA was shown to reverse airway hyperresponsiveness (AHR) in an *Aspergillus fumigatus *mouse model of asthma [88]. Similar results were also reported for TSA in an OVA mouse model of asthma, with reduced AHR, lower lymphocytic and eosinpohilic infiltrate in the BALF, as well as reduced IL-4, IL-5, and IgE levels in the BALF [89].

## 7. Histone Deacetylase Inhibitors and Cancer Immunotherapy

Altered HDAC function and recruitment could result in enhanced hypoacetylation and repression of genes required for normal growth and development and disruption of the HDAC/HAT balance favouring abnormal acetylation and inappropriate protein expression could provide the molecular trigger that directs pathological outcomes [5, 90, 91]. The discovery that HDAC function is dysregulated in cancer and has a pivotal role in tumourigenesis justifies the use of HDAC inhibitors as an effective approach to restore this balance [92].

An exciting area of cancer research has focused on the therapeutic efficacy of HDACi in modulating antitumour immunity. Of the many diverse biological HDACi activities, recent evidence suggests that tumour immunosurveillance may be enhanced by direct manipulation of tumour cells or indirectly via changes in the immune microenvironment [1]. Such immunological niches could provide enhanced regulation of tumour growth and development. Several studies have shown that tumour antigenicity is enhanced following HDACi treatment, thereby preventing tumour escape. HDACi such as sodium butyrate, TSA, and trapoxin A among others upregulate MHC class I and class II proteins, CD40, CD80, and CD86 antigens necessary for costimulation, as well as adhesion molecules such as intercellular adhesion molecule-1 (ICAM-1) on acute myeloid leukemic (AML) cells, human neuroblastoma tumour cells, and mouse plasmacytoma cells *in vitro *[93, 94]. These results suggest an important function of HDACi in tumour eradication. Moreover, TSA, SAHA, VPA, and sodium butyrate were also found to increase the expression of the MHC class I-related chain A (MICA) and chain B (MICB) on tumour cells [95, 96]. The MICA and MICB proteins are ligands for Natural Killer (NK) group 2, member D (NKG2D) activating receptors on NK cells, CD8+ T cells, and *γδ* T cells important for the immune-targeted destruction of tumour cells. In the case for TSA, the mechanisms for this tumour cytotoxicity were associated with increased histone H3 acetylation and reduced HDAC1 expression at the MICA and MICB promoters regions [97]. Furthermore, the immunogenicity of epithelial tumour cells was increased by TSA via upregulating UL16-binding protein (ULBP) expression (an NKG2D ligand) and enhancing NK cell-mediated tumour cytotoxicity related to the release of HDAC3 from the ULPB promoter [98]. Enhanced tumour cell cytotoxicity also occurs following RNAi-mediated HDAC11 inhibition, increasing OX40L expression in Hodgkin lymphoma cell lines associated with elevated TNF-*α* and IL-17 levels in the supernatant [99]. In contrast with other HDACi, inhibition of HDAC11 in this study lowered IL-10-producing Treg numbers which favoured the elimination of these tumour cells. Taken together, this suggests that HDACi exhibit a variety of effects based on the disease context and their tissue and cellular specificity.

HDACi have also been successful in the prevention and treatment of cancer through therapeutic vaccination. Treatment with TSA or depsipeptide augmented the expression of the cancer vaccine target, Cancer/Germ-line family of antigens (CG antigens), as well as MHC and costimulatory molecules on tumours, facilitating immune system targeting [100]. In addition, TSA also enhanced the expression of MHC class II, CD40, and B7-1/2 antigens on B16 melanoma cells that resulted in the induction of IFN-*γ*-secreting CD8+ T cells and NK cells [101]. However, the mechanism(s) by which HDACi augment tumour clearance is not fully understood. Contrasting these HDACi functions, HDACi such as TSA were implicated in assisting tumour escape from the immune system by decreasing the presentation and killing of tumour cells by CD8+ T cells [102].

Given the immunomodulatory properties of HDACi described previously, enhanced antitumour immunity could also result from altered cytokine profiles. Suppression of the proinflammatory cytokines, IL-1, TNF-*α*, and IFN-*γ* has been reported in studies of allogeneic bone marrow transplantation [55] possibly via epigenetic changes at the promoter/enhancer regions of cytokine genes as well as regulatory transcription factors (e.g., STAT1, 3) [1]. An example of this was observed for the HDACi, SAHA, by altering the Th1/Th2 cytokine balance favouring Th1 by inhibiting STAT6-mediated IL-5 production and secretion of Thymus and Activation-Regulated Chemokine (TARC or CCL17) as well as increasing the levels of IL-10 and IL-13 in Hodgkin lymphoma cell lines *in vitro *[103].

## 8. New Generation Histone Deacetylase Inhibitors

The mechanism by which HDAC inhibitors mediate the diverse antitumour activities is not fully understood. While the pan-HDACi, SAHA and panobinostat (LBH-589), are effective against many different tumours and are generally well-tolerated, recent evidence suggests that more tumour-specific reagents are required [104–107]. The accumulating data on HDAC expression in tumours will allow researchers to develop particular isoform-specific candidates with increased efficacy. For instance, in haematological cancers, HDAC1, 2, and 6 predominate while for solid tumours, HDAC expression is variable with HDAC1-3 (gastric and colorectal cancer), HDAC1, 4–7, 10 (liver cancer) and HDAC1, 3, 6 (breast cancer) described [108]. More specific compounds may modulate a smaller number of genes specific for a particular disease as well as exhibiting a lower toxicity profile compared to the reported 22% of genes said to be regulated by pan-HDACi [32, 109]. The level of HDAC expression can also be thought of as a biomarker for certain responsive tumour types in addition to the acetylation status of histone proteins during clinical trials of HDACi. However, the identification of novel biomarkers that can predict the response to treatment may be more beneficial in targeting particular HDACi in specific cases [110].

The requirement for more specific and efficacious anticancer treatments has led to implementation of natural product screening programs to identify novel compounds that exhibit such properties. Naturally occurring compounds are a rich resource for potential new drug candidates [111]. Similar to the National Cancer Institute's extensive natural product screening programs last century that provided the medical community with the now commonly used anticancer drugs paclitaxel, vincristine, and vinblastine, new generation HDACi have also been identified from nature. In particular, compounds derived from the rhizomes of *Zingiber zerumbet, *a south-east Asian ginger, and the roots of *Pleuropterus ciliinervis *exhibited HDAC-associated growth inhibitory activity on several human tumour cell lines [112, 113]. Pomiferin isolated from the fruits of *Maclura pomifera *could also inhibit growth of human tumour cells but was lower than that for SAHA [114]. While structural similarities of these novel compounds to classical HDACi such as SAHA were apparent, this may not necessarily be sufficient to predict HDACi activity, as one such compound was found to possess no such inhibitory activity towards ARP-1 human myeloma cells *in vitro *[115].

## 9. Conclusions and Future Directions

The therapeutic potential of HDACi offers an alternative approach to the treatment of a wide variety of conditions such as cancer and immune-mediated diseases. The pleiotropic effects of HDACi include inhibition of cancer cell proliferation and differentiation as well as inducing proapoptotic and antiangiogenesis events. In relation to the immunomodulatory properties of HDACi described in this paper, epigenetic regulation of histone and nonhistone transcription factors influence important pathways for the generation of effective immune responses. Moreover, HDACi prevent the ability of tumours to evade the immune system by enhancing host immunosurveillance and inducing appropriate local immune effector functions. The search for more specific compounds from sources such as plants may unearth highly efficacious next-generation HDACi of clinical importance.
